# Exploring the influence of sagittal imbalance on spinal mobility, function and quality of life in axial spondyloarthritis: a novel comprehensive compensatory mechanism of adaptation

**DOI:** 10.1186/s42358-025-00495-y

**Published:** 2025-11-24

**Authors:** Thauana Luiza de Oliveira, Flávio Duarte Silva, Alípio Gomes Ormond Filho, Marcelo Astolfi Caetano Nico, Artur da Rocha Correa Fernandes, Sofia Ramiro, Marcelo de Medeiros Pinheiro

**Affiliations:** 1 Present Address: Rheumatology Division, Federal University of São Paulo/Escola Paulista de Medicina (UNIFESP/EPM), São Paulo, Brazil; 2 Diagnostic Imaging Department, Federal University of São Paulo/Escola Paulista de Medicina (UNIFESP/EPM), São Paulo, Brazil; 3 Diagnostic Imaging Department, Fleury Medicina e Saúde, São Paulo, Brazil; 4 Department of Rheumatology, Leiden University Medical Center, the Netherlands and Zuyderland Medical Center, Heerlen, the Netherlands; 5 Present Address: Rheumatology Division, Rua Borges Lagoa, 913/ 53, Vila Clementino, São Paulo, CEP 04038-034 SP Brazil

**Keywords:** Axial spondyloarthritis, Postural balance, Patient reported outcome measures

## Abstract

**Objectives:**

To investigate whether sagittal imbalance, defined by sagittal vertical axis (SVA) using EOS^®^ imaging, is associated with spinal mobility, function, and quality of life in patients with axial spondyloarthritis (axSpA).

**Methods:**

Patients with axSpA were cross-sectionally assessed for sagittal imbalance (SVA ≥ 50 mm). Spinal mobility (BASMI), function (BASFI) and quality of life (ASQoL) were compared between patients with and without sagittal imbalance. Multivariable analyses examined the associations between SVA and the above-mentioned disease outcomes, adjusted for confounders. Mediation analysis explored whether sagittal alignment mediated the relationship between spinal mobility and structural damage.

**Results:**

Among 117 patients (mean age 51 (SD 11) years, 68% males), 44 (38%) had sagittal imbalance. SVA was only independently and significantly associated with BASMI but not with BASFI or ASQoL. SVA minimally mediated the relationship between mSASSS and BASMI. The optimal BASMI cutoff to identify patients with sagittal imbalance was 5.2 with 80% of correct classification.

**Conclusions:**

Sagittal imbalance is associated with impaired spinal mobility but not with impaired function or quality of life. These findings may reflect compensatory mechanisms for sagittal balance in long-term axSpA patients. Impaired spinal mobility can be used to identify patients with sagittal imbalance who may benefit from physiotherapy or rehabilitation.

**Clinical trial number:**

Not applicable.

## Key points

- Sagittal imbalance is not associated with function or quality of life in ax-SpA.
- Only 4.7% of the effect of mSASSS on BASMI was mediated by sagittal vertical axis.
- The optimal BASMI cutoff to identify patients with sagittal imbalance was 5.2.

## Introduction

The concept of spinopelvic balance, also called sagittal balance, is related to the ideal spinal positioning parameters in the sagittal plane, resulting from the interaction between the spine and the lower limbs, with the pelvis working as a link between them [1, 2].

EOS^®^ (Biospace, Paris, France) is a device that allows simultaneous acquisition of coronal and sagittal radiographic images of the spine with a low radiation dose. The patient stands in a functional orthostatic position and whole-body images are obtained through a single scan with two orthogonal X-ray beams. Through EOS^®^ an important sagittal balance parameter can be obtained, the sagittal vertical axis (SVA). Sagittal balance refers to ideal spinal alignment parameters, whereas sagittal imbalance can be defined by an SVA ≥ 50 mm [3–6].

In clinical practice there are some conditions that can lead to spinal deformities and thus require compensatory mechanisms to be activated to maintain sagittal balance, both at the spinal and pelvic levels or in the lower limbs, as is the case for patients with degenerative disc disease, scoliosis, facet joint osteoarthritis, spondylolysis, and osteoporosis with vertebral fractures [7–10]. Patients with axial spondyloarthritis (axSpA), particularly radiographic axSpA, earlier known as ankylosing spondylitis, who have developed increasing thoracolumbar kyphosis over the years may suffer sagittal balance changes, compromising their ability to look at the horizon and to perform various activities in daily life and work [11–14]. Throughout this process, the center of gravity tends to move anteriorly. In an attempt to rectify this situation, there is a progressive retroversion of the pelvis and hip extension. However, this adaptation mechanism may be insufficient to completely compensate for the development of thoracolumbar kyphosis, which could cause pain, fatigue, and increased energy expenditure [15]– [16].

An association between sagittal imbalance and reduced quality of life has been previously shown in patients with degenerative lumbar kyphoscoliosis and osteoporosis [7–10]. However, there are only a few studies evaluating the relationship between sagittal balance and clinical outcomes in patients with axSpA, but none of them have simultaneously analyzed parameters of spinal mobility, function and quality of life [17–20]. Recently, our group has shown that more spinal structural damage is associated with a higher SVA in patients with axSpA, reflecting poorer sagittal balance [21].

Our aim was to investigate the potential association between SVA and relevant disease outcomes among patients with axSpA. We hypothesized that axSpA patients in sagittal imbalance, defined as SVA ≥ 50 mm, would have worse spinal mobility, compromised function and impaired quality of life.

## Methods

### Patient selection

In this cross-sectional study, patients with a diagnosis of axSpA and fulfilling the Assessment of SpondyloArthritis International Society (ASAS) classification criteria for radiographic axial spondyloarthritis were consecutively recruited at the outpatient clinic of Spondyloarthritis at the Federal University of São Paulo/ Escola Paulista de Medicina (UNIFESP/ EPM – Brazil) from July 2018 to November 2019 [22]. Eligibility criteria included a symptom onset age of 18 years or older, stable pharmacological treatment for the past six months and no hip or knee prosthesis. Patients with non-radiographic axial spondyloarthritis were excluded, as their inclusion would predominantly represent cases without structural damage or spinal deformity, thereby reducing the likelihood of sagittal imbalance within the study sample. The study was approved by the Ethics Committee of Federal University of São Paulo (CAAE: 80660517.7.0000.5505) in accordance with the Declaration of Helsinki, and all patients signed the informed consent form upon participation.

### AxSpA relevant outcome measures

All patients underwent examination by the same rheumatologist (TLO), who collected demographic, clinical, laboratory, anthropometric data and on disease outcomes, including assessment of disease activity (Axial Spondyloarthritis Disease Activity Score – ASDAS), functional impairment (Bath Ankylosing Spondylitis Functional Index – BASFI), and quality of life (Ankylosing Spondylitis Quality of Life - ASQoL) [23–25]. In addition, spinal mobility of patients was measured using the linear definition of Bath Ankylosing Spondylitis Metrology Index (BASMI) [26, 27]. For BASFI and BASMI, the score ranges from 0 to 10, with 10 being related to marked functional and mobility impairment, respectively [24, 26]. For ASQoL, the score is from 0 to 18, with lower values being related to better quality of life [25].

Cervical and lumbar spine conventional radiographs were independently evaluated by two readers (TLO and MMP) using the modified Stoke Ankylosing Spondylitis Spinal Score (mSASSS), as previously described [21, 28].

### Sagittal balance measurement

**Fig. 1:**
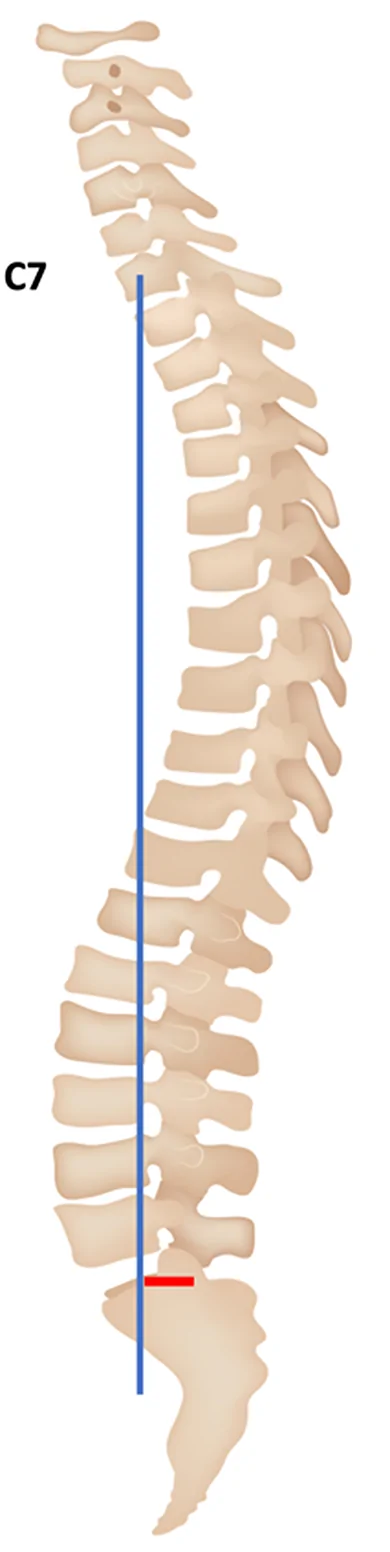
Schematic representation of sagittal vertical axis (SVA) which is indicated by the red line. SVA represents the distance in millimeters between a plumb line dropped from the center of the cervical (C)7 vertebra and the posterior superior corner of the sacral plateau. The SVA measurement provides information about the overall spine sagittal balance. One of the key criteria for defining sagittal imbalance is an SVA measurement greater than or equal to 50mm

Sagittal balance was measured through SVA, which was obtained using the EOS^®^. SVA is defined as the distance from a plumb line from the center of cervical (C)7 vertebra until the posterior superior corner of the sacral plateau (Fig. 1). The patient was positioned in an orthostatic position, with the feet spaced 20–25 cm apart and the hands resting on a crossbar positioned in front of the body and at the height of the iliac crest. The sterEOS^®^ workstation was used for the review, manipulation, and storage of images in Digital Imaging and Communications in Medicine (DICOM) format. The two-dimensional radiographic images were subsequently analyzed by a biomedical technician who had received training from a radiologist experienced in EOS^®^ imaging (FD). He identified the centers of the sacral plateau, femoral heads, and C7 vertebra to enable the automatic calculation of the SVA by EOS^®^. SVA was calculated in the patient plane (vertical plane passing through the center of acetabulum), which corrects for the effect of a possible axial rotation of pelvis at time of image acquisition [3].

One of the primary indicators used to define sagittal imbalance is an SVA measurement surpassing 50 mm. Values lower than 50 mm have been linked to a better quality of life and reduced disability compared with higher SVA levels in individuals with adult spinal deformities [5–7]. We therefore divided our population into two groups according to the SVA value: group 1 (SVA < 50 mm) and group 2 (SVA ≥ 50 mm).

### Statistical analysis

For the categorical variables, the absolute and relative frequencies are presented, and for the continuous variables, mean, standard deviation (SD). The data normality was inspected with histograms and using the Kolmogorov‒Smirnov test. Continuous variables were compared using two-sample t-test for normally distributed variables or Mann‒Whitney test for non-normally distributed variables. Univariable linear regression analysis was performed to evaluate the relationship between SVA and the above-mentioned axSpA outcome measures. Subsequently, multivariable linear regression analysis was conducted, with Model 1 incorporating SVA as a continuous variable, and Model 2 treating it as a dichotomous one. The assumptions of linear regression (linearity, homoscedasticity, Independence, and normal distribution of residuals) were verified, and multicollinearity was ruled out based on variance inflation factors below 5. No missing data were identified for the variables included in the analysis. The independent variables sex, age, BMI, human leukocyte antigen (HLA)-B27, body mass index (BMI), ASDAS and mSASSS were also included as potential confounders in the model when BASMI and ASQoL were the dependent variables. BASFI and BASMI were each included in the model when ASQoL and BASFI were used as the dependent variables, respectively. Considering the potential relationship between mSASSS and BASMI could be mediated by SVA, we developed two models with BASMI as the dependent variable: Model 1 (including mSASSS) and Model 2 (excluding mSASSS). Variables not contributing to explain the outcome, nor confounding the relationship of main interest were removed using backward selection. A mediation analysis using the Sobel test and Bootstrapping was conducted to explore whether the relationship between BASMI and mSASSS was mediated by SVA [29]. Additionally, to determine the BASMI threshold associated with an increased likelihood of sagittal imbalance, a Receiver Operating Characteristic (ROC) analysis was performed and the best performing cutoff was chosen using four methods: (1) Percentile 75 of BASMI: this method calculates the 75th percentile of the BASMI scores as a cutoff point for distinguishing patient groups; (2) Liu Method: a statistical approach optimizing the trade-off between sensitivity and specificity to minimize misclassification error; (3) Youden index: calculated as the sum of sensitivity and specificity minus one, this index identifies the cutoff that maximizes the balance between true positives and false positives and (4) Proximity to coordinate (0,1) of the ROC curve: this method looks for the point on the ROC curve that is closest to the top-left corner (0,1), indicating perfect sensitivity and specificity, to maximize diagnostic accuracy [30].

Significant level was set as below 5%. The statistical software packages SPSS 20.0 were utilized for data analysis.

### Role of the funding source

This study was funded by grants from Brazilian Society of Rheumatology and Fleury Medicina e Saúde, which covered the costs of EOS^®^ imaging exams.

## Results

A total of 117 patients was included, with a mean age of 51 (11) years and 68% were men. In total, 44 (38%) patients were in sagittal imbalance, i.e. SVA ≥ 50 mm. Comparing patients with and without sagittal imbalance, there was no difference between the groups regarding age, HLA-B27, or BMI, with a higher prevalence of men and longer disease duration in the group with sagittal imbalance (SVA ≥ 50 mm). In addition, this group also had higher BASFI, BASMI and mSASSS and there was no difference between the groups considering disease activity or quality of life (Table 1). Approximately 70% of patients in both groups were receiving anti–tumor necrosis factor (anti-TNF) (49/73 in group 1 and 31/44 in group 2). No patients were using other classes of biologic agents.

**Table 1:** Demographic and disease characteristics of included patients with axial spondyloarthritis, according to the sagittal vertical axis

	Group 1, sagittal balance (SVA < 50 mm) (*N* = 73)	Group 2, sagittal imbalance (SVA ≥ 50 mm) (*N* = 44)	*p*-value
Age (years)	48 (11)	52 (10)	0.11
Disease duration (years)	19 (9)	23 (8)	**0.02**
Sex (male), n (%)	42 (57)	37 (84)	**0.01**
HLA-B27+, n (%)	58 (79)	33 (75)	0.76
BMI (kg/m^2^)	28.04 (4.51)	26.97 (4.17)	0.28
ASQoL (0–18)	6.44 (5.85)	6.75 (5.94)	0.99
ASDAS	2.09 (0.98)	2.10 (0.81)	0.70
BASMI (0–10)	3.10 (2,83)	4.83 (2.79)	**0.008**
BASFI (0–10)	3.44 (2.11)	6.10 (2.18)	**< 0.001**
mSASSS (0–72)	10.57 (14.09)	35.74 (20.66)	**< 0.001**

Data are presented as mean (standard deviation, SD) unless otherwise specified. SVA (sagittal vertical axis); HLA (Human leukocyte antigen); BMI (Body Mass Index); ASQoL (Ankylosing Spondylitis Quality of Life); ASDAS (Axial Spondyloarthritis Disease Activity Score); BASMI (Bath Ankylosing Spondylitis Metrology Index); BASFI (Bath Ankylosing Spondylitis Functional Index), mSASSS (modified Stoke Ankylosing Spondylitis Spine Score)

In the univariable model, we observed that a higher SVA was associated with higher BASMI and BASFI values, thus worse outcomes. The same was found when analyzing the dichotomous SVA (SVA > = 50 vs. < 50 mm).

After adjusting for confounders, SVA was only independently and significantly associated with BASMI: a 10 mm elevation in SVA was associated with an average increase of 0.1 points in the BASMI (Table 2). Sagittal imbalance itself (i.e. SVA ≥ 50 mm) was also associated with higher BASMI scores (regression coefficient 1.08 (95% CI 0.17;1.98)). HLA-B27 positivity was associated with better spinal mobility, while older age and a higher mSASSS were associated with a worse spinal mobility. In the multivariable models without mSASSS, the relationship between SVA and BASMI was slightly stronger and a higher ASDAS was associated with a worse spinal mobility.

**Table 2:** Relationship between sagittal balance and spinal mobility (multivariable analysis)

	Multivariable regression (model with mSASSS)				Multivariable regression (model without mSASSS)			
	Adjusted coefficient (95%CI) Model1 – SVA as continuous variable *N* = 117	*p*	Adjusted coefficient (95%CI) Model 2 – SVA as a dichotomous variable *N* = 117	*p*	Adjusted coefficient (95%CI) Model1 – SVA as continuous variable *N* = 117	*p*	Adjusted coefficient (95%CI) Model 2 – SVA as a dichotomous variable *N* = 117	*p*
Sagittal vertical axis (SVA) (mm)	0.01 (0.007 to 0.02)	**< 0.001**	&		0.03 (0.02 to 0.04)	**< 0.001**	&	
Sagittal vertical axis (SVA) dichotomized (reference = < 50 mm)	&		1.08 (0.17 to 1.98)	**0.01**	&		1.14 (0.52 to 1.76)	**< 0.001**
Sex (reference = female)	0.39 (-0.39 to 1.19)	0.32	0.48 (-0.34 to 1.30)	**0.24**	0.42 (-0.36 to 1.22)	0.28	0.53 (-0.26 to 1.33)	0.18
Age (years)	0.03 (0.004 to 0.07)	**0.02**	0.04 (0.006 to 0.07)	0.02	0.06 (0.02 to 0.09)	**< 0.001**	0.06 (0.02 to 0.09)	**< 0.001**
BMI (kg/m^2^)	0.00 (-0.08 to 0.07)	0.95	0.00 (-0.07 to 0.08)	0.87	-0.01 (-0.09 to 0.06)	0.67	-0.01 (-0.09 to 0.06)	0.67
HLA-B27+	-0.87 (-1.66 to -0.09)	**0.02**	-0.76 (-1.58 to -0.05)	0.06	-0.87 (-1.6 to -0.13)	**0.02**	-0.80 (-1.54 to -0.06)	**0.03**
ASDAS	0.29 (-0.07 to 0.66)	0.11	0.32 (-0.06 to 0.70)	0.10	0.35 (0.01 to 0.68)	**0.04**	0.31 (-0.02 to 0.65)	0.07
mSASSS (0–72)	0.04 (0.02 to 0.07)	**< 0.001**	0.05 (0.02 to 0.07)	**< 0.001**	&		&	

& Variable not included in the model. SVA (sagittal vertical axis); BMI (Body Mass Index); HLA (Human leukocyte antigen); ASDAS (Axial Spondyloarthritis Disease Activity Score), mSASSS (modified Stoke Ankylosing Spondylitis Spinal Score)

There was no association between SVA or sagittal imbalance with ASQoL or BASFI. A higher BASFI was associated with a higher ASQoL (worse quality of life), while a higher ASDAS and BASMI were both associated with a higher BASFI (worse function) (Table 3).

**Table 3:** Relationship between sagittal balance and function (BASFI) and quality of life (ASQoL) (multivariable analysis)

	Multivariable regression (BASFI)				Multivariable regression (ASQoL)			
	Adjusted coefficient (95%CI) Model1 – SVA as continuous variable *N* = 117	*p*	Adjusted coefficient (95%CI) Model 2 – SVA as a dichotomous variable *N* = 117	*p*	Adjusted coefficient (95%CI) Model1 – SVA as continuous variable *N* = 117	*p*	Adjusted coefficient (95%CI) Model 2 – SVA as a dichotomous variable *N* = 117	*p*
Sagittal vertical axis (SVA) (mm)	0.00 (-0.01 to 0.014)	0.78	&		0.00 (-0.03 to 0.03)	0.99	&	
Sagittal vertical axis (SVA) dichotomized (reference = < 50 mm)	&		0.03 (-1.15 to 1.22)	0.95	&		-0.90 (-3.57 to 1.17)	0.5
Sex (reference = female)	-0.25 (-1.32 to 0.80)	0.62	-0.25 (-1.31 to 0.80)	0.63	-1.07 (-3.46 to 1.31)	0.37	-1.08 (-3.46 to 1.29)	0.36
Age (years)	-0.00 (-0.04 to 0.04)	0.96	0.00 (-0.04 to 0.04)	0.95	0.00 (-0.09 to 0.10)	0.93	0.00 (-0.10 to 0.10)	0.98
BMI (kg/m^2^)	0.06 (-0.04 to 0.16)	0.24	0.06 (-0.04 to 0.16)	0.24	0.03 (-0.19 to 0.27)	0.74	0.03 (-0.19 to 0.27)	0.73
HLA-B27+	0.90 (-0.16 to 1.97)	0.09	0.91 (-0.15 to 1.97)	0.09	-0.16 (-2.60 to 2.27)	0.89	-0.19 (-2.61 to 2.23)	0.87
ASDAS	1.29 (0.79 to 1.79)	**< 0.001**	1.29 (0.78 to 1.79)	**< 0.001**	1.39 (0.11 to 2.68)	**0.03**	1.35 (0.06 to 2.64)	**0.03**
BASMI (0–10)	0.56 (0.28 to 0.85)	**< 0.001**	0.57 (0.29 to 0.84)	**< 0.001**	-0.19 (-0.88 to 0.5)	00.58	-0.14 (-0.81 to 0.53)	0.67
BASFI (0–10)	&&		&		1.43 (0.94 to1.91)	**< 0.001**	1.43 (0.95 to 1.91)	**< 0.001**
mSASSS (0–72)	0.00 (-0.02 to 0.03)	0.73	0.00 (-0.02 to 0.04)	0.73	-0.01 (-0.09 to 0.05)	0.64	-0.00 (-0.08 to 0.06)	0.83

& Variable not included in the model. ASQoL (Ankylosing Spondylitis Quality of Life); SVA (sagittal vertical axis); BMI (Body Mass Index); HLA (Human leukocyte antigen); ASDAS (Axial Spondyloarthritis Disease Activity Score); BASMI (Bath Ankylosing Spondylitis Metrology Index); BASFI (Bath Ankylosing Spondylitis Functional Index), mSASSS (modified Stoke Ankylosing Spondylitis Spinal Score)

The mediation analysis indicated that the indirect effect of mSASSS on BASMI, mediated by SVA (both continuous and dichotomized), was statistically significant but very small, practically negligible (4.7% of indirect effect).

**Fig. 2:**
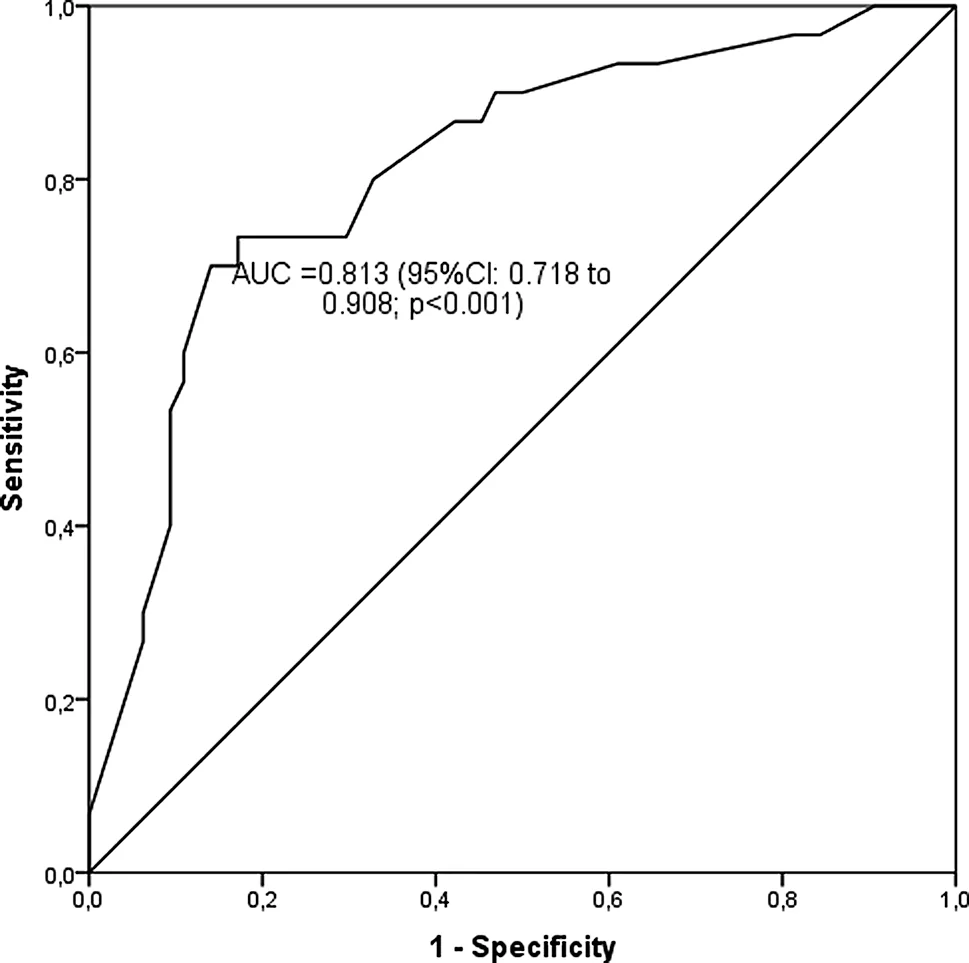
Receiver Operating Characteristic curve for defining sagittal imbalance (SVA≥50 mm) based on spinal mobility (BASMI). AUC was 0.813 (95% CI 0.718-0.908, *p*<0.001). SVA (sagittal vertical axis); BASMI (Bath Ankylosing Spondylitis Metrology Index); AUC (area under the curve); CI (Confidence interval)

As shown in Table 4, the cut-off point identified by Youden’s criterion, Liu’s method, and proximity to the point (0,1) was 5.2 and this demonstrated higher sensitivity, negative predictive value, and a lower negative likelihood ratio compared to the 75th percentile of BASMI. A BASMI ≥ 5.2 correctly classified 80% of patients with sagittal imbalance (SVA ≥ 50 mm) with a sensitivity of 73%, a specificity of 83% and an area under the curve of 0.813 (Fig. 2).

**Table 4:** Sensitivity, specificity, predictive values and positive and negative likelihood ratios of different cut-off points of BASMI

Method	Cut-off point	Sensitivity (95% CI) (%)	Specificity (95% CI) (%)	Percentage of correct classification	Predictive Value (95% CI)		Likelihood Ratio (95% CI)	
					Positive	Negative	Positive	Negative
Youden, Liu and nearest to 0.1	5.2	73.3 (54.1–87.7)	82.8 (71.3–91.1)	79.8%	66.7 (48.2–82)	86.9 (75.8–94.2)	4.3 (2.4–7.6)	0.3 (0.2–0.6)
P75 – BASMI	6.4	56.7 (37.4–74.5)	89.1 (78.8–95.5)	78.7%	70.8 (48.9–87.4)	81.4 (70.3–89.7)	5.2 (2.4–11.1)	0.5 (0.3–0.7)

CI (confidence interval); P75 (Percentile 75); BASMI (Bath Ankylosing Spondylitis Metrology Index)

## Discussion

To our knowledge, this is the first study demonstrating the relationship between BASMI and sagittal balance, as measured with SVA, even when adjusted for mSASSS. Although the increase in BASMI score is numerically small for each 10 mm rise in SVA, considering that mobility is influenced by multiple factors beyond the structural damage resulting from axSpA, this finding adds further insight into the complex relationship between structural damage, range of motion and sagittal imbalance. Recently, our group has reported the relationship between structural radiographic damage, as assessed by mSASSS, and SVA. Specifically, each one-unit increase in total mSASSS was associated with an average 0.8 mm higher SVA [21]. Our mediation analysis shows that the effect of mSASSS on BASMI is not mediated by SVA. These results illustrate the complexity of spinal postural alterations that occur in axSpA when the patient progresses with radiographic damage. Compensatory mechanisms, such as the capacity for hip extension (which might be reduced in patients with secondary hip osteoarthritis due to the disease), and paravertebral muscle strength and mass, which can impact overall posture, may also play a role in this compensatory process [31, 32]. Additional studies are warranted to determine the clinical relevance of these findings and to elucidate the role of compensatory mechanisms in this process. We did not observe a significant association between sagittal balance and the axSpA disease outcomes of quality of life and function.

As previously shown, spinal mobility and radiographic spinal damage are directly associated, but spinal mobility cannot be relied upon as a sole proxy for radiographic damage assessment [33]. Similarly, the BASMI alone may not be sufficient to identify patients with sagittal imbalance, which can be assessed more accurately using imaging techniques such as EOS^®^. However, the BASMI may help to identify patients at higher risk of developing such postural alterations, who could consequently benefit from a referral for physiotherapy and rehabilitation [34, 35].

Disease activity (ASDAS) rather than sagittal balance (SVA) contributed to explain both quality of life and functional impairment, reinforcing that controlling the inflammatory activity of the disease is essential for patients to maintain their quality of life and function, as widely known [36–38]. Furthermore, functional impairment was associated with quality of life and in turn, both disease activity and spinal mobility were also associated with functional impairment, in line with what was previously known [39].

In clinical practice, patients with long-standing axSpA often develop adaptive strategies to maintain stability, function, and quality of life despite severe mobility impairment and structural damage. These adaptations include postural adjustments, use of assistive devices, muscle strengthening, and avoidance of activities that exacerbate pain or inflammation. Psychological and emotional support also fosters resilience and adherence to treatment. Although difficult to quantify, it is possible that these coping mechanisms may influence the relationship between sagittal balance (SVA) and disease outcomes in axSpA [40].

This study has some limitations such as the cross-sectional design, which precludes the inference of causal relationships between structural damage, mobility, and sagittal imbalance, and lack of a healthy control and non-radiographic ax-SpA groups. In addition, the identification of landmark markings for EOS^®^ measurements was carried out by a single assessor. Besides that, we did not examine the radiographic damage to the hips, which could affect disease outcomes, especially mobility, as well as sagittal imbalance. Notwithstanding, this was the first study to measure sagittal balance through EOS^®^ and examine the potential impact of SVA and sagittal imbalance on multiple disease outcomes in patients with axSpA.

## Conclusions

In conclusion, our study shows that sagittal imbalance is associated with worse spinal mobility, while structural damage is associated with both. Moreover, despite this impact, unlike what is observed in other spinal deformity diseases, sagittal imbalance does not independently contribute to functional disability or impairment in quality of life, possibly due to compensatory mechanisms.

## Data Availability

The data can be made available from authors upon request.
